# Impulsivity and social support as intervening and interactive variables in the link between childhood socioeconomic status and mental health among first-year college students

**DOI:** 10.3389/fpsyt.2025.1569001

**Published:** 2025-04-17

**Authors:** Yiting Kong, Zhewei Su, Rui Wang, Jianyu Tan, Pan Ran, Xiaoming Xu, Wo Wang, Su Hong, Qi Zhang, Li Kuang

**Affiliations:** ^1^ Department of Psychiatry, The First Affiliated Hospital of Chongqing Medical University, Chongqing, China; ^2^ Psychiatric Center, The First Affiliated Hospital of Chongqing Medical University, Chongqing, China; ^3^ Mental Health Center, University-Town Hospital of Chongqing Medical University, Chongqing, China

**Keywords:** childhood socioeconomic status, mental health, impulsivity, social support, first-year college students

## Abstract

**Background:**

Significant attention has been given to the mental health of college students, especially first-year college students, with childhood socioeconomic status (SES) identified as a key factor. This study investigated the correlation of impulsivity and social support in the relationship between childhood SES and current mental health, with a focus on depressive and anxiety symptoms, in first-year college students.

**Methods:**

A cross-sectional study was designed, surveying 6,378 first-year students (mean age = 20.98) at a university in Chongqing, China. The survey participants were 63.4% female and 36.6% male. The participants completed an online questionnaire which included Patient Health Questionnaire-9, Generalized Anxiety Disorder-7, Brief Barratt Impulsiveness Scale, Multidimensional Scale of Perceived Social Support, and a 7-point scale to measure childhood SES. Descriptive statistics and correlation analyses were conducted for these variables, and the data examined further using a moderated conditional effect model with PROCESS macro (Model 8).

**Results:**

The analysis revealed that lower childhood SES showed small to moderate negative correlations with impulsivity (r = -0.224, p < 0.01, small effect) and heightened symptoms of depression (β = -0.235, p < 0.01) and anxiety (β = -0.197, p < 0.01). Impulsivity shared variance with the link between childhood SES and both depressive (β = 0.386, SE = 0.011, p < 0.001) and anxiety symptoms (β = 0.315, SE = 0.012, p < 0.001). Higher levels of social support were linked to attenuated associations between low childhood SES and both impulsivity (β = -0.064, SE = 0.011, p < 0.01) and depressive symptoms (β = -0.029, SE = 0.010, p < 0.01). However, social support was not significantly associated with the link between childhood SES and anxiety symptoms.

**Conclusion:**

Our findings demonstrate that impulsivity serves as a partial intervening variable in the relationship between childhood SES and the mental health of first-year college students. However, higher levels of social support were linked to weaker negative associations between impulsivity and both childhood SES and mental health. Interventions that focus on managing impulsivity and increasing social support for first-year college students from low socioeconomic backgrounds could be effective strategies for improving their mental health.

## 1 Introduction

The psychological well-being of college students, who constitute a vital demographic for societal progress, has garnered substantial attention due to its escalating prevalence and multifaceted repercussions. Empirical data reveal that mental health challenges extend beyond emotional distress: American College Health Association estimates that over 75% of undergraduates experience mental health difficulties, predominantly anxiety and depression (1), while approximately 28.4% of Chinese university students exhibit clinically relevant depressive symptoms (2). These issues are exacerbated by transitional stressors unique to early university life. First-year college students face a perfect storm of transitional stressors that uniquely predispose them to mental health crises. Neurodevelopmental immaturity—marked by incomplete prefrontal cortex regulation of limbic reactivity (3)—converges with the abrupt collapse of childhood social networks (4) and exposure to rigid academic hierarchies (5), driving diagnosable psychological conditions in 33% of freshmen (6), often stemming from academic overload, social adaptation challenges, and uncertainty about future trajectories (3, 4).

The consequences permeate multiple domains: impaired academic performance (5), disrupted sleep patterns (7), and heightened risks of social withdrawal (8). Critically, untreated psychological distress correlates with a 1.8-fold increase in dropout likelihood (9), perpetuating cycles of educational and occupational disadvantage (10). These findings collectively underscore the urgency of prioritizing mental health interventions during the vulnerable first-year transition, where students navigate intersecting pressures that amplify susceptibility to chronic stress and maladaptive coping (11, 12).

The causes and mechanisms of mental health problems are highly complex. Adversities in early childhood are recognized as a high-risk factors (13). Research has shown that socioeconomic adversities can impact the physical and mental health of succeeding generations through intergenerational transmission (14). Socioeconomic status (SES), one of the most studied concepts in social science, is usually characterised by family income, parental education level, and occupational status. Previous studies have shown that childhood SES has extensive effects on the health, cognition, and the emotional well-being of a person, the effects of which may well over into adulthood (15, 16). According to the social causation theory, SES impacts child development through mechanisms such as the family stress model and the family investment model (17). Multiple studies have consistently found a link between childhood SES and mental health challenges in later life, specifically depression and anxiety disorders (18–20). A more recent study showed that SES during childhood negatively affected the mental health of college students (21). Given this context, it is essential to explore the link between childhood SES and mental health, as well as statistical associations, specifically among college students.

Impulsivity, a multifaceted construct encompassing emotion-driven reactivity and impaired inhibitory control (22, 23), emerges as a transdiagnostic vulnerability bridging childhood socioeconomic adversity and internalizing symptoms through both evolutionary and neurocognitive mechanisms. Life-history theory posits that early-life resource scarcity fosters impulsive strategies prioritizing immediate stress reduction over long-term planning (24)—a behavioral constellation of deprivation that becomes maladaptive in stable environments (25), perpetuating cycles of socioeconomic stress (26). Neurobiologically, chronic stress from low childhood SES disrupts prefrontal-striatal circuitry (27), impairing top-down regulation of limbic-driven emotional reactivity (28, 29). This manifests behaviorally as impulsive responses to negative affect (30), where individuals oscillate between rash action (e.g., procrastination amplifying academic failure anxiety) and emotional avoidance (e.g., suppressing distress until it erupts as pathological rumination)—patterns strongly associated with depressive and anxious symptomatology (31, 32). However, the detrimental effects of impulsivity may not be inevitable. Social support, by providing resources for stress buffering and adaptive coping (12), could disrupt the pathway from childhood SES to impulsivity and subsequent mental health decline, as observed in retention programs targeting at-risk students (9).

Social support is defined as the perceived or actual provision of instrumental and/or expressive resources provided by the community, social networks, and trusted individuals (33). Scholars have categorized social support into three primary categories: support from family, support from friends, and support from significant others (34). According to the classic main effect model and the stress-buffering model (35), social support is believed to not only directly fosters positive psychological states and maintain individual mental health, but also mitigates the occurrence of psychological problems by buffering against stress and reducing its responses. Previous studies have consistently acknowledged the positive impact of strong social support in preserving mental health, mitigating stress, and lowering the incidences of depressive symptoms (36). Research indicates that college students with lower levels of social support are more susceptible to mental health challenges, particularly, depressive symptoms (37).

Previous research has also reported that positive interpersonal interactions can help mitigate the adverse effects of low SES, including poverty and adversity, on both physical and mental health (38). This goes to show that a strong social support system can be linked to weaker the negative associations between low SES on mental health. Additionally, social support has been recognized as a mitigating factor against risky behaviors, such as drug use and abuse, among college students (39), which are often associated with impulsivity (40, 41). Social support thus not only benefits mental health but may also help protect against the development of impulsivity.

**Figure 1:**
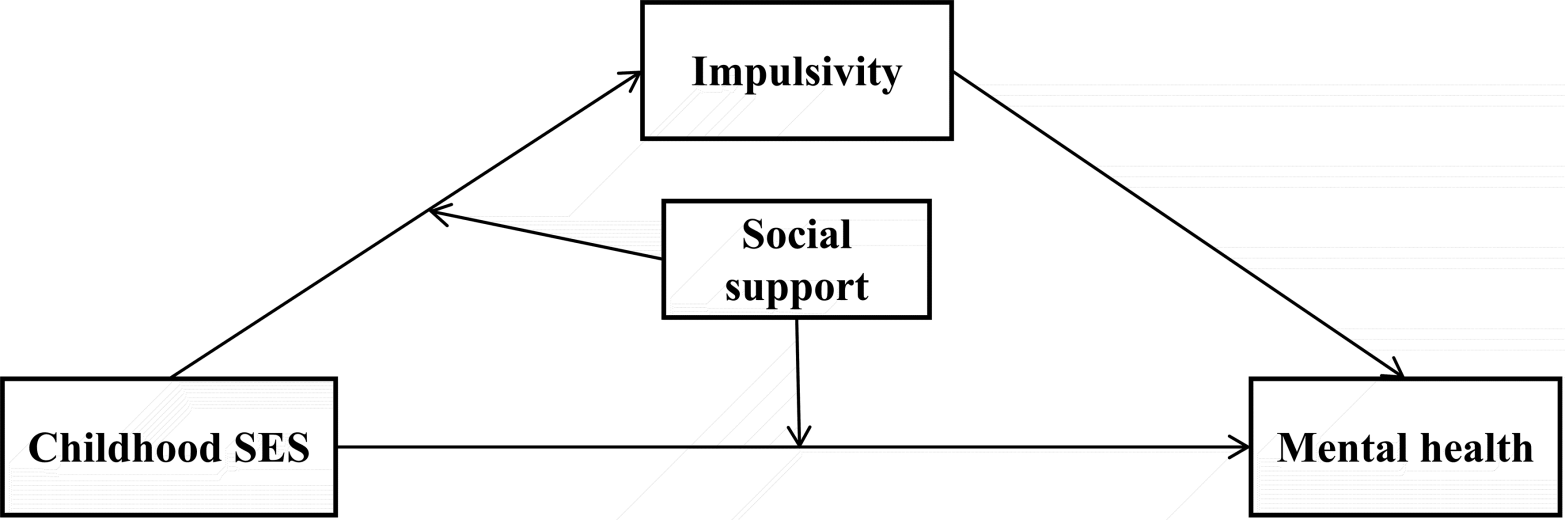
Hypothesized moderated conditional effect model. SES, socioeconomic status.

Existing research has extensively examined the links between childhood SES, mental health, impulsivity, and social support across diverse populations. However, critical gaps persist in understanding these relationships among first-year college students. While prior studies often analyze these factors in isolation, few have holistically modeled their dynamic interactions, limiting our capacity to design targeted interventions for this vulnerable transitional population. To bridge these gaps, this study targeted first-year college students at one university in China, with a focus on how impulsivity and social support correlate with the relationship between childhood SES and mental health issues. Building on evidence that childhood SES influences impulsivity through chronic stress pathways (2), and that impulsive traits amplify vulnerability to mental health challenges (11), we further propose social support as a contextual buffer. Specifically, social support may mitigate both the direct effects of low SES and its indirect effects via impulsivity. A moderated conditional effect model was designed to shed more light on the correlation of childhood SES on mental health problems (**Figure 1**). This model also focused on the intervening correlation of impulsivity and the contextual correlation of social support. Based on the hypothesized model, this study proposed the following three hypotheses:

*Hypothesis 1:* Childhood SES is related to the mental health of first-year college students, with lower childhood SES correlating with more pronounced depressive and anxiety symptoms.
*Hypothesis 2:* Impulsivity shares variance with the connection between childhood SES and mental health. Specifically, lower childhood SES is linked to increased levels of impulsivity, which subsequently exacerbates symptoms of depression and anxiety.
*Hypothesis 3:* Social support may attenuate the observed associations between childhood SES and mental health, both directly and through its statistical linkage with impulsivity. Specifically, higher social support is associated with less pronounced negative relationships between lower childhood SES and mental health, as well as fewer depressive and anxiety symptoms concurrent with a weaker childhood SES-impulsivity connection.

## 2 Materials and methods

### 2.1 Participants

A survey on mental health among first-year students at Chongqing Medical University in China was conducted in collaboration with the school authorities in September 2023. Sampling from a single university allowed us to control institutional-level confounders (e.g., academic policies, campus resources) and focus on individual-level mechanisms (e.g., childhood SES, impulsivity). Chongqing, as a major urban center in Southwest China, reflects socioeconomic dynamics common to rapidly developing regions. While not fully representative of rural or ethnic minority areas, this setting provides insights into mental health challenges faced by students in high-pressure academic environments.

The survey employed an electronic questionnaire asking the sociodemographic characteristics, depression, anxiety, childhood SES, impulsiveness and social support. Approximately 20 min was needed to complete the questionnaire. All participants were informed of the contents of this survey and their consent obtained through a consent statement attached at the beginning of the questionnaire’s cover page, where only those who pressed the “agree” button could participate in the survey. Non-participation in this survey would not adversely affect students’ academic standing. Participants who withdrew or did not complete the questionnaires (n = 281) were excluded. An additional 39 participants were removed due to patterned answers and excessive short responses. Ultimately, a total of 6,378 participants, including first-year college and master’s students aged 18 to 25, were included in the final analysis.

The study protocol was reviewed and approved by the Ethics Committee of the National Clinical Medical Research Center at the Second Xiangya Hospital, Central South University (Approval No. 2022S010), which served as the lead ethics committee for this multi-center study. Chongqing Medical University, as a participating site, fully complied with the ethical guidelines and procedures authorized by the lead committee. All procedures adhered to pertinent guidelines and regulations, including the Declaration of Helsinki.

### 2.2 Measures

#### 2.2.1 Mental health

The Patient Health Questionnaire-9 (PHQ-9) (42) and the Generalized Anxiety Disorder-7 (GAD-7) (43) were utilized to assess the participants’ mental health, by their depressive and anxiety symptoms.

PHQ-9 consists of nine items assessing depressed mood, low motivation, sleep, appetite, and suicidal thoughts. The total score of all items on PHQ-9 were used to reveal the severity of the depression, with 5 or greater representing the cut-point for identifying depression (42). GAD-7 is a seven-item scale assessing feelings of anxiety, restlessness and irritability. The aggregate score from all items on the GAD-7 scale was used to measure the severity of anxiety symptoms. Total score of 5 or greater represents a cut-point for identifying anxiety (43).

Both PHQ-9 and GAD-7 use a 4-point Likert scale (0 = not at all, 1 = several days, 2 = more than half the days, 3 = nearly every day) to reveal the participants’ mental status over the previous 2 weeks (42, 43). These 2 scales were validated in Chinese (44, 45). The cumulative scores from these scales were computed, with higher scores on the questionnaires representing more severe symptoms of depression or anxiety respectively. The Cronbach’s α coefficient for the PHQ-9 and GAD-7 were 0.857, 0.903 respectively.

While originally developed for clinical settings, the PHQ-9 and GAD-7 have been widely validated for use in student populations. A cutoff score of ≥5 indicates mild symptoms, which are common in high-stress academic environments. However, as transient stressors (e.g., exams) may temporarily elevate scores, this study focuses on symptom severity rather than clinical diagnoses, reporting prevalence as “mild to severe symptoms” rather than classifying participants as clinical cases.

#### 2.2.2 Childhood socioeconomic status

To assess participants’ childhood SES, a 7-point scale developed by Griskevicius et al. (46), anchored from 1 “strongly disagree” to 7 “strongly agree,” was utilized. The scale has three items: (a) “My family usually had enough money for things when I was growing up”; (b) “I grew up in a relatively wealthy neighborhood”; (c) “I felt relatively wealthy compared to the other kids in my school” (46). The scale was validated by Griskevicius et al. (46) as a concise yet reliable measure of subjective childhood SES in large-scale studies. It captures three core dimensions of SES: family financial security, neighborhood wealth, and relative social standing during childhood. The total score of this scale was calculated and with the low scores indicating poor childhood SES. The Cronbach’s α was 0.894.

#### 2.2.3 Impulsivity

Participants’ Impulsivity was assessed using the Brief Barratt Impulsiveness Scale (BBIS) (47). BBIS uses a 4-point Likert scale (1 = never, 2 = rarely, 3 = sometimes, 4 = often) containing two dimensions (poor self-regulation and impulsive behavior) and eight items. Items 1, 4, 5, 6 are reverse scoring. The total score of all items was calculated, with higher scores indicating stronger impulsivity. The Cronbach’s α for BBIS was 0.776.

#### 2.2.4 Social support

Multidimensional Scale of Perceived Social Support (MSPSS) (34), which has good reliability, was used to assess the social support of all participants. MSPSS uses a 7-point psychometrical instrument, anchored at “strongly disagree” and “strongly agree”. Support from family, friends and significant others was measured on a 3-subscale structure of the MSPSS. The total score of all 12 items was calculated to assess the social support of participants. Higher total scores indicated greater social support. The Cronbach’s α for MSPSS was 0.954.

### 2.3 Data analysis

Statistical analyses were carried out using SPSS 26.0 software. Descriptive statistics, including mean and standard deviation (SD), were calculated. Pearson’s correlation analysis was conducted to examine the relationships among variables, and independent samples t-tests were performed to assess gender differences in depressive and anxiety symptoms. To minimize potential multi-collinearity, the primary variables were standardized before analysis (48). The relationships between variables were analysed using the PROCESS macro in SPSS (Models 4 and 8) to test the hypotheses (49). The macro used 5000 bootstraps samples to estimate the model and calculate 95% confidence intervals (95% CI) (50). Relationships were deemed significant if the 95% CI did not include 0. The significance level was established at α=0.05, with gender and age considered as covariates. Beta coefficients (β) reported in our path models are standardized regression coefficients. These values represent the change in the outcome variable (in standard deviation units) per one-standard-deviation increase in the predictor variable, adjusted for covariates.

To address potential common method bias arising from self-reported data, we conducted Harman’s single-factor test (51). The first unrotated factor was associated with 30.29% of the total variance, below the 50% threshold, indicating that common method bias did not significantly correlate with our results (51, 52).

### 2.4 Priori power analysis

Prior to data collection, we conducted *a priori* power analysis using Fritz and MacKinnon’s Monte Carlo method for conditional effects (53). Assuming a small-to-moderate indirect effect (a×b = 0.08, based on meta-analytic estimates of childhood SES and mental health relationships), a significance level of α = 0.05, and 80% power, the required sample size was estimated to be N = 1,200. Our final sample (N = 6,378) exceeded this threshold, ensuring robust detection of hypothesized effects.

### 2.5 Transparency and reproducibility

We report how we determined our sample size, all data exclusions, all manipulations, and all measures in this study. Sample size determination was based on institutional enrollment and power analysis. Data exclusions were based on predefined criteria for response quality, including patterned responses and excessively short completion times. No experimental manipulations were applied, as this study used observational survey data. All measures used in the study are described in detail in the Materials and methods section.

## 3 Results

### 3.1 Descriptive analyses

As shown in **Table 1**, data from 6,378 participants were considered, with 4,043 of them being women (63.4%) and 2,335 men (36.6%) was analyzed. The average age was 20.98 years (SD = 3.37). Of the 6,378 participants, 2,181 (34.20%) exhibited mild to severe depressive symptoms (Total score of PHQ-9 ≥ 5), while 2,349 students (36.83%) exhibited mild to severe anxiety symptoms (Total score of GAD-7 ≥ 5). Pearson’s correlation analysis identified a positive correlation between age and both depressive (r = 0.087, p < 0.001) and anxiety symptoms (r = 0.089, p < 0.001). Additionally, independent samples t-tests further revealed significant gender differences in depressive symptoms (t = -6.95, p <0.001) and anxiety symptoms (t = -7.16, p <0.001). Specifically, female participants reported higher levels of depressive symptoms (Female: 5.03 ± 3.56; Male: 4.36 ± 3.94) and anxiety symptoms (Female: 3.82 ± 3.35; Male: 3.19 ± 3.44) compared to male participants. Based on these findings, including gender and age as covariates statistically adjusted for the baseline disparity ensured that observed effects were not attributable to these factors.

**Table 1:** Demographic characteristics of participants.

Variables		N/Mean	%/Standard
Age		20.98	3.37
Gender	Female	4043	63.40
	Male	2335	36.60
Academic Level	First-year college students	4266	66.89
	First-year master’s students	2112	33.11
Ethnicity	Han Chinese	5825	91.33
	Ethnic Minority	553	8.67
Parental Marital Status	Married and harmonious	5134	80.50
	Separated or Divorced	1244	19.50
Mother’s education	Primary school or below	1663	26.07
	Secondary school or associate degree	3386	53.09
	Bachelor’s degree or above	1329	20.84
Father’s education	Primary school or below	1141	17.89
	Secondary school or associate degree	3608	56.57
	Bachelor’s degree or above	1629	25.54
PHQ-9	≥5	2181	34.20
	< 5	4197	65.80
GAD-7	≥5	2349	36.83
	< 5	4029	63.17

N =6378. PHQ-9 = Patient Health Questionnaire-9; GAD-7 = Generalized Anxiety Disorder-7.

### 3.2 Correlational analyses

The bivariate correlations among all variables were examined using Pearson’s correlation analysis (**Table 2**). Consistent with Cohen’s criteria (54), childhood SES demonstrated a small to moderate negative correlation with impulsivity (r = -0.224, p < 0.01) and small inverse associations with depressive (r = -0.235) and anxiety symptoms (r = -0.197). Furthermore, impulsivity exhibited a positive correlation with both depressive symptoms (r = 0.439, moderate effect) and anxiety symptoms (r = 0.362, moderate effect).

**Table 2:** Descriptive statistic and correlations among the variables.

Variables	Mean	SD	1	2	3	4	5
1.Depressive symptoms	4.79	3.71	–				
2.Anxiety symptoms	3.59	3.39	0.764^**^	–			
3.Childhood SES	15.62	5.92	-0.235^**^	-0.197^**^	–		
4.Impulsivity	15.71	3.99	0.439^**^	0.362^**^	-0.224^**^	–	
5.Social support	67.80	13.09	-0.389^**^	-0.341^**^	0.347^**^	-0.332^**^	–

N =6378. SD, standard deviation; SES, socioeconomic status.

^**^ *p* < 0.01.

To explore these associations further, a multivariate linear regression analysis was conducted (**Table 3**). Variance Inflation Factors (VIFs) ranged from 1.01 to 2.64, below the threshold of 5, indicating acceptable collinearity. The results indicated that higher childhood SES was significantly associated with lower levels of depressive (β = -0.153, p < 0.001) and anxiety symptoms (β = -0.130, p < 0.001), after controlling for gender and age. Additionally, impulsivity was positively associated to both depressive (β = 0.386, p < 0.001) and anxiety symptoms (β = 0.315, p < 0.001).

**Table 3:** Results from the multiple linear regression analysis examining the relationship between childhood SES, impulsivity and mental health.

	Depressive symptoms				Anxiety symptoms			
	Unadjusted		Adjusted^%^		Unadjusted		Adjusted%	
	β	t	β	t	β	t	β	t
Childhood SES	-0.144^***^	-12.630	-0.153^***^	-13.582	-0.123^***^	-10.317	-0.130^***^	-11.054
Impulsivity	0.407^***^	35.690	0.386^***^	34.013	0.334^***^	28.152	0.315^***^	26.593

SES,= socioeconomic status.

*%* Adjusted for gender and age.

***p < 0.001.

### 3.3 Testing for conditional effect

Childhood SES emerged as a significant predictor of depressive (β = -0.242, SE = 0.012, p < 0.001) and anxiety symptoms (β = -0.203, SE = 0.012, p < 0.001) after gender and age were controlled (**Table 4**). With the introduction of impulsivity as a intervening variable, childhood SES remained a significant predictor for depressive (β = -0.153, SE = 0.011, p < 0.001) and anxiety symptoms (β = -0.130, SE = 0.012, p < 0.001). Further analysis of the conditional effect model revealed that childhood SES could significantly predict impulsivity (β = -0.231, SE = 0.012, p < 0.001), which in turn, was a significant predictor of both depressive (β = 0.386, SE = 0.011, p < 0.001) and anxiety symptoms (β = 0.315, SE = 0.012, p < 0.001).

**Table 4:** Conditional effect model test.

Variables	Depressive symptoms -Direct Model			Depressive symptoms - Conditional Effect Model			Impulsivity			Anxiety symptoms -Direct Model			Anxiety symptoms - Conditional Effect Model		
	β	SE	t	β	SE	t	β	SE	t	β	SE	t	β	SE	t
Gender	0.158	0.025	6.404***	0.151	0.023	6.650***	0.018	0.025	0.727	0.167	0.025	6.686***	0.162	0.024	6.805***
Age	-0.195	0.012	-16.402***	-0.142	0.011	-12.805***	-0.139	0.012	-11.481***	-0.172	0.012	-14.242***	-0.128	0.012	-11.072***
Childhood SES	-0.242	0.012	-20.329***	-0.153	0.011	-13.582***	-0.231	0.012	-19.084***	-0.203	0.012	-16.815***	-0.130	0.012	-11.054***
Impulsivity				0.386	0.011	34.013***							0.315	0.012	26.593***
R2	0.099			0.237			0.069			0.075			0.167 319.883***		
F	233.387***			495.996***			158.489***			171.750***					

SES, socioeconomic status; Direct Model, the model without the intervening variable (impulsivity); Conditional Effect Model, the model including impulsivity as an intervening variable.

***p<0.001.

The proportion analysis indicated that impulsivity accounted for 36.78% of the shared variance in the association between childhood SES and depressive symptoms and 35.96% in the association with anxiety symptoms (**Table 5**). While a significant portion of the effect remained direct, these findings suggest that impulsivity plays an important role in the relationship between childhood SES and mental health outcomes.

**Table 5:** Proportion of indirect effects of impulsivity.

	Depressive symptoms				Anxiety symptoms			
	Effect	SE	95% CI	Effect Ratio	Effect	SE	95% CI	Effect Ratio
Total effect	-0.242	0.012	[-0.266, -0.219]		-0.203	0.012	[-0.227, -0.203]	
Direct effect	-0.153	0.011	[-0.175, -0.153]	63.22%	-0.130	0.012	[-0.153, -0.107]	64.04%
Indirect effect	-0.089	0.006	[-0.100, -0,078]	36.78%	-0.073	0.005	[-0.083, -0.063]	35.96%

SE, standard error; 95% CI, 95% confidence interval.

### 3.4 Testing for moderated conditional effect

**Figure 2:**
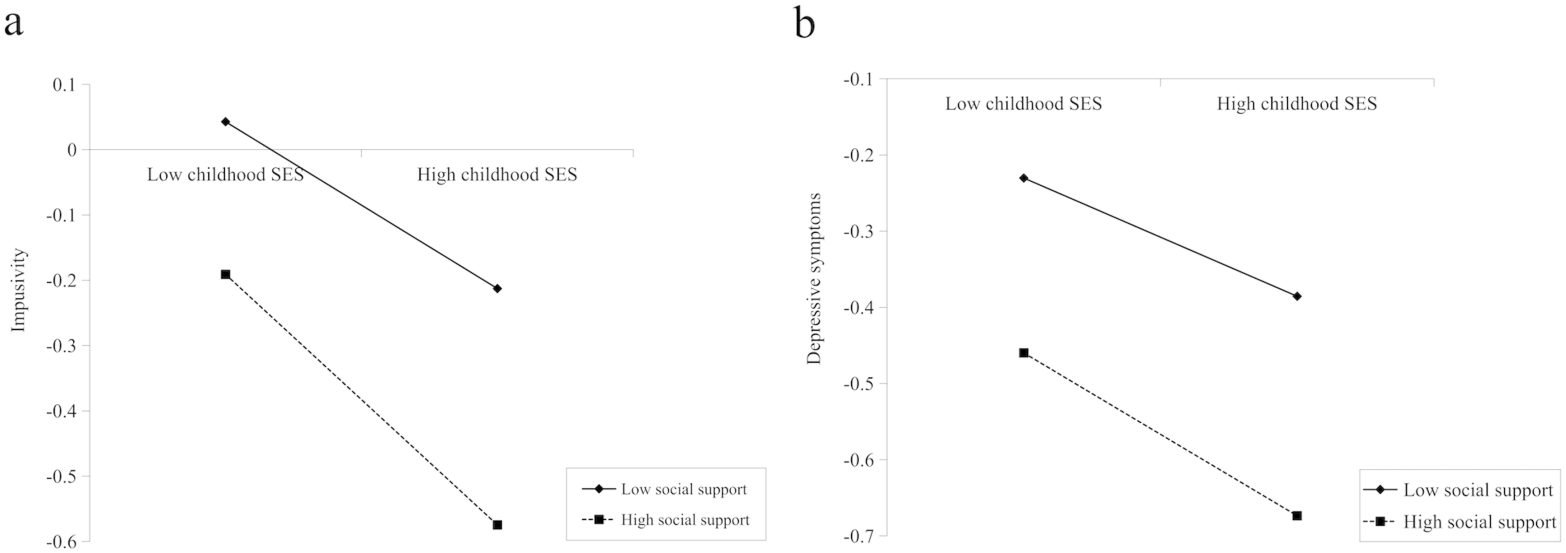
Simple Slopes Plot for **(a)** impulsivity and **(b)** depressive symptoms.

**Figure 3:**
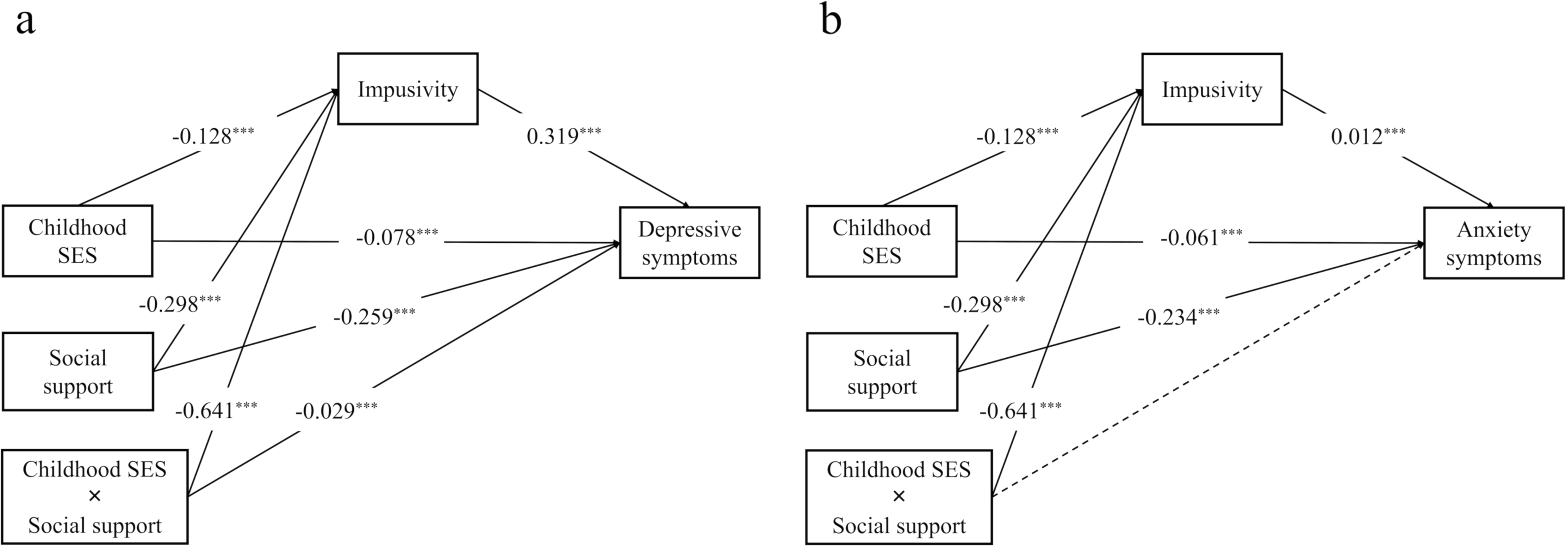
Moderated conditional effect model of **(a)** depressive, and **(b)** anxiety symptoms SES, socioeconomic status ^***^ *p* < 0.001.

After adjusting for covariates, all significant pathways identified in the conditional effect model persisted. Specifically, childhood SES significantly predicted depressive (β = -0.078, SE = 0.012, p < 0.001) and anxiety symptoms (β = -0.061, SE = 0.012, p < 0.001), and impulsivity (β = -0.128, SE = 0.013, p < 0.001). Impulsivity remained a significant predictor of depressive (β = 0.319, SE = 0.011, p < 0.001) and anxiety symptoms (β = 0.255, SE = 0.012, p < 0.001). Moreover, social support was a significant predictor of impulsivity (β = -0.298, SE = 0.013, p < 0.001), depressive symptoms (β = -0.259, SE = 0.012, p < 0.001), and anxiety symptoms (β = -0.234, SE = 0.013, p < 0.001). The interaction between childhood SES and social support significantly predicted impulsivity (β = -0.064, SE = 0.011, p < 0.01) as well as depressive symptoms (β = -0.029, SE = 0.010, p < 0.01). A simple slope analysis as shown in **Figure 2**, indicated that higher social support levels mitigated the association between childhood SES, with both impulsivity and depressive symptoms. However, this interaction did not significantly predict anxiety symptoms (**Table 6**, **Figure 3**, and **Figure 2**).

**Table 6:** Moderated and conditional effect analysis.

	Impulsivity			Depressive symptoms			Anxiety symptoms		
	β	SE	t	β	SE	t	β	SE	t
Gender	0.066	0.024	2.710^***^	0.195	0.022	8.822^***^	0.202	0.023	8.678^***^
Age	-0.107	0.012	-9.158^***^	-0.124	0.011	-11.520^***^	-0.112	0.011	-9.890^***^
Childhood SES(A)	-0.128	0.013	-10.228^***^	-0.078	0.012	-6.774^***^	-0.061	0.012	-5.073^***^
Social support(B)	-0.298	0.013	-23.109^***^	-0.259	0.012	-21.181^***^	-0.234	0.013	-18.143^***^
A × B	-0.064	0.011	-5.899^***^	-0.029	0.010	-2.951^***^	-0.006	0.010	-0.584
Impulsivity				0.319	0.011	27.930^***^	0.255	0.012	21.194^***^
R^2^	0.141			0.288			0.210		
F	209.859^***^			429.968^***^			282.503^***^		

***p<0.001.

## 4 Discussion

Research suggests that first-year university students face heightened stress, increasing risks of psychosocial maladjustment, especially for those with lower childhood SES (3, 6, 17, 30). However, social support has been shown to buffer stress and enhance coping, mitigating these effects (35, 37). Building on these findings, this study focused on first-year college students, exploring the potential relationships among childhood SES, mental health, impulsivity, and social support.

This study demonstrates that lower childhood SES is directly linked to heightened depressive and anxiety symptoms among first-year college students, with impulsivity serving as a partial conditional effector. Social support emerged as a protective buffer, attenuating the SES-depressive symptoms pathway but not anxiety symptoms. Notably, female students reported higher depressive and anxiety symptom severity compared to males, aligning with global patterns of internalizing disorders disproportionately affecting women (6, 11). These findings align with life-history theory, where early-life resource scarcity fosters impulsive coping strategies to manage immediate stressors (25), and the stress-buffering hypothesis, which posits that social support mitigates chronic stress (35). The results extend prior work by integrating neurobiological and cultural perspectives unique to Chinese students (55, 56).

These results fully support Hypothesis 1. Longitudinal studies consistently link low childhood SES to adult depression and anxiety through chronic stress pathways (57, 58). Another study targeting university students highlighted a positive correlation between childhood subjective SES and their mental well-being in college (21). Exposure to low SES during childhood may be linked to increased chronic physiological stress. This may manifest itself through heightened sympathetic nervous system activity, elevated hypothalamic-pituitary-adrenal (HPA) axis activity, metabolic dysregulation, and more severe immune-inflammatory responses, eventually leading to physical and mental health issues (59–62).

This chronic stress may also manifest behaviorally as impulsivity—a dysregulation of inhibitory control mechanisms (3, 63, 64)—which prior studies associate with both socioeconomic adversity and heightened mental health risks (2, 11). A study examining various ethnic groups have reported that even after controlling for race and gender, higher family income at birth was lined to lower impulsivity during adolescence (65). Our findings corroborate these patterns and highlight impulsivity as a novel conditional effector. For example, structural MRI study confirms that low SES correlates with reduced prefrontal cortex volume, a neural substrate for self-regulation (66). And lower SES is associated with blunted HPA axis reactivity and prefrontal-striatal circuit dysregulation (66, 67), which impair inhibitory control and amplify impulsive behaviors (68). These mechanisms align with Evans and Kim’s model of socioeconomic deprivation and self-regulation deficits (59).

Impulsivity, often conceptualized as dysregulated self-control (23), is associated with the SES-mental health link by exacerbating emotion-driven reactivity (30). This aligns with studies showing that early adversity disrupts executive functions and increases vulnerability to internalizing disorders (69, 70). Numerous studies have supported the notion that heightened impulsivity is a risk factor for conditions such as depression and anxiety (68, 70). This study similarly found that higher impulsivity is associated with more severe depressive and anxiety symptoms among first-year college students. Additionally, previous research has confirmed that impulsivity can share variance with the relationship between adverse experiences in childhood and psychological and behavioral problems (such as self-harm, dissociative symptom) in adulthood (71, 72). A recent systematic review also suggested that impulsivity could share variance with the relationship between childhood maltreatment and adult suicidal behavior (73). Together with the findings in this study, these results confirm Hypothesis 2, indicating that increased impulsivity may be a potential mechanism through which childhood SES impacts the mental health of first-year college students.

The bootstrap analysis from Model 8, further revealed that social support moderates the relationship between childhood SES and impulsivity, as well as the connection between childhood SES and depressive symptoms. These findings partially support Hypothesis 3, suggesting that social support helps reduce the rise in impulsivity associated with lower childhood SES and as well as lessening the direct effects of childhood SES on symptoms of depression in first-year college students. Social support is widely accepted as being closely linked to mental health (74, 75) and is regarded to be a vital protective element for the mental health of college students (37, 56). There is extensive research showing that social support can alleviate the effects of negative childhood experiences, including those of childhood SES on psychological and behavioral challenges (76–78). Other studies across different populations have also found that social support helps to reduce the detrimental effects of various risk factors on depressive symptoms (79–81). For instance, research on Chinese adolescents and young adults revealed that social support and optimism can share variance with the relationship between SES and depression (55). Additionally, recent research has shown that emotional support from parents can be instrumental in modulating various aspects of impulsivity in early adulthood (81). From an immuno-inflammatory perspective, social support can lessen the immune inflammation triggered by chronic stress associated with low childhood SES (82, 83), This in turn reduces the negative effects of adverse childhood experiences, decreasing impulsivity and depressive symptoms as well. Furthermore, from a stress-response perspective, research has demonstrated that social support can lower cortisol responses to stress, providing a buffering effect that reduces an individual’s reaction to chronic stress (84, 85). In light of the findings from this study, the importance of social support in preventing high impulsivity and depressive symptoms is further emphasized.

The observed relationships between childhood SES, impulsivity, and mental health may be interpreted within the cultural context of Chinese society. In China, family cohesion and collectivist values often prioritize interdependence and filial piety, which may amplify the role of social support as a protective factor against mental health challenges. For instance, familial support in Chinese culture is not merely emotional but also encompasses material and instrumental assistance (e.g., financial aid, academic guidance) (55), which may be associated with why social support significantly buffered the SES-depression pathway in our study.

This study did not however identify a significant direct moderating effect of social support on the pathway from childhood SES to anxiety symptoms. Previous research on university student has reported the protective role of social support against anxiety (86–88). Several reasons could be behind this inconsistency between the findings of this study and previous research. First and foremost, the results of this study are not entirely contradictory to past findings. Although the direct moderating effect on anxiety symptoms was not observed, increased social support was shown to reduce anxiety symptoms indirectly through its impact on impulsivity. This suggests a potential mechanism by which it functions as a protective factor against anxiety symptoms. Secondly, the differing tools which were used to measure social support and anxiety symptoms in this study (MSPSS for social support and GAD-7 for anxiety) compared to those used in previous research may not fully capture the nuanced aspects of social support relevant to anxiety, resulting in the moderating effect being less significant in this case. Third, in collectivist cultures like China, stigma around mental health may limit social support’s efficacy for anxiety (56), which requires targeted interventions. Furthermore, depressive symptoms and anxiety symptoms, though often comorbid, have distinct psychological mechanisms. Depression is closely linked to feelings of hopelessness and low self-worth, which may be more directly alleviated by perceived social support (e.g., reassurance of self-value through interpersonal connections) (74). In contrast, anxiety often involves hypervigilance to perceived threats (89), which may be less responsive to generalized social support and require more targeted interventions. Future studies should explore the differential effects of distinct types of social support (e.g., emotional support vs. instrumental support) on anxiety symptoms to elucidate their specific mechanisms. Finally, since the participants in this research consisted exclusively of first-year college students, differences in sample characteristics may account for the variations observed.

### 4.1 Strengths and limitations

To the best of our knowledge, this is groundbreaking research in examining how impulsivity and social support correlated with the link between childhood SES and mental health among first-year college students. To assess the reliability of the observed conditional effects, we conducted a *post-hoc* power analysis using Fritz and MacKinnon’s Monte Carlo method (53). This approach estimated the statistical power for detecting the indirect effect based on our observed path coefficients. The calculated power exceeded 99% for both paths (depressive symptoms and anxiety symptoms), indicating that our sample size provided ample sensitivity to detect even small conditional effects. The results offer important practical implications. First, the correlation of SES on children’s healthy development should be emphasized, with more support provided to children to prevent potential mental health issues in adulthood. Second, universities could prioritize the establishment of structured psychological counseling services tailored to the unique needs of students from disadvantaged backgrounds. For instance, embedding mandatory mental health screenings during orientation programs may facilitate early identification of at-risk individuals, allowing for timely referrals to counseling resources. Peer mentorship initiatives, where upperclassmen with similar socioeconomic experiences guide incoming students, could reduce stigma and foster trust in seeking help. Such programs have demonstrated efficacy in enhancing social connectedness and academic retention (12). Additionally, collaborations with community mental health organizations could expand access to subsidized therapy, addressing financial barriers that disproportionately affect low-SES students. Third, for the prevention and intervention of psychological problems, more emphasis should be placed on impulse control training, such as social and emotional learning (SEL) programs (90), should be emphasized to alleviate symptoms of depression and anxiety. Workshops grounded in SEL principles, such as modules on emotional regulation and decision-making, could be integrated into first-year seminars. Academic advisors could further reinforce these skills by incorporating practical tools—such as time management frameworks and goal-setting exercises—into routine advising sessions, bridging the gap between behavioral strategies and academic success. Finally, students identified with risk factors should receive targeted interpersonal support and be helped to establish and develop social support systems during their university years in order to safeguard their mental health development. Universities might develop cohort-based programs that group first-year students into small communities led by faculty advisors, fostering peer bonding and resource-sharing. Family engagement initiatives, such as workshops educating parents on mental health challenges and their role in providing emotional support, could strengthen external support systems.

Despite the important findings of this study, there are limitations that should be acknowledged. First, the data analyzed in this study were collected through online self-report questionnaires, which may carry the risk of subjective bias. Thus, researchers should formulate more detailed questionnaire designs (for instance, factors that were not explored in this study such as living arrangements, which may influence the psychosocial adjustment of first-year college students) combined with structured interviews to further investigate the complex relationships among variables. Second, the findings of this study are derived from a single university in China, which may limit their applicability to populations in different cultural or educational settings. For instance, socioeconomic challenges and mental health stigma may manifest uniquely in other regions or educational systems. However, this focused sampling allowed us to deeply explore the mechanisms linking childhood SES, impulsivity, and mental health within a homogeneous cohort of first-year students, thereby reducing confounding effects of institutional variability. Future multi-center studies across diverse geographic and cultural contexts are warranted to validate these findings and assess their broader relevance. Third, the gender imbalance in our sample may limit generalizability to gender-balanced populations. Future research should validate these findings in cohorts with equitable gender representation and explore potential moderating effects of gender on impulsivity, social support, and mental health linkages. Fourth, the BBIS captures only two facets of impulsivity (self-regulation deficits and impulsive behavior), omitting cognitive and non-planning dimensions. Future research should employ multidimensional instruments like the BIS-11 to disentangle impulsivity subtypes and enhance mechanistic insights. Fifth, while linear regression models (e.g., PROCESS) assume additive relationships, psychological constructs may exhibit nonlinear or interactive dynamics. Future studies could employ nonlinear regression or structural equation modeling (SEM) to explore complex pathways, particularly gene-environment interactions or threshold effects. Sixth, another limitation pertains to the measurement of childhood SES, which relied on retrospective self-reports. This approach may introduce recall bias, as participants’ recollections of early-life socioeconomic conditions could be correlated with current mental states or subjective interpretations. To enhance measurement reliability in future research, we recommend incorporating objective indicators of childhood SES, such as parental education levels, household income records, or archival data (e.g., neighborhood socioeconomic indices). These multi-method approaches would provide a more comprehensive and less biased assessment of socioeconomic status during childhood. Seventh, while all participants were first-year students, the age range (18–25 years) may introduce variability due to developmental or experiential differences. For instance, older students (e.g., ≥22 years) might have delayed enrollment due to gap years or academic setbacks, potentially possessing greater coping skills or facing distinct socioeconomic pressures. Although we statistically controlled for age in our analyses, residual confounding may persist. This heterogeneity may limit the generalizability of findings to typical first-year students (aged 18–19). Future studies should stratify analyses by age subgroups or adopt longitudinal designs to disentangle age-related effects from cohort-specific transitions.

Finally, the cross-sectional design inherently constrains the interpretative scope of our single-center study, primarily manifested in three dimensions of methodological ambiguity. First, the inability to establish temporal precedence precludes causal inferences regarding the developmental pathway from childhood SES through impulsivity to mental health outcomes, given that current psychological states (e.g., depressive symptoms) may systematically bias retrospective SES evaluations while simultaneously shaping social support perceptions—a dual-directional confounding mechanism well-documented in developmental psychopathology (91, 92). Second, our observed 36.78% conditional effect of impulsivity on depressive symptoms, though statistically robust through bootstrap validation, likely conflates between-person trait differences (e.g., chronic impulsivity) with within-process dynamics, as cross-sectional models intrinsically overestimate indirect effects compared to longitudinal designs (91). Third, despite controlling for age and gender, residual confounding persists from unmeasured genetic factors (e.g., dopamine receptor polymorphisms affecting both impulsivity and stress reactivity) and dynamic environmental stressors (e.g., academic pressures during data collection)—limitations mitigated yet not eliminated by our multi-covariate adjustment approach. Nevertheless, our hypothesized pathways are supported by longitudinal evidence. For instance, childhood SES effects on impulsivity are mechanistically explained by chronic stress and neurodevelopmental disruptions (61), and impulsivity’s role in exacerbating mental health symptoms is well-documented (68). Moreover, despite methodological constraints, our findings are statistically robust and align with longitudinal evidence. For example, the indirect effect of impulsivity (36.78%) mirrors effect sizes reported in multi-wave studies (65), suggesting our cross-sectional results may approximate true longitudinal relationships. To enhance the reliability of these findings and establish causal relationships, future studies should adopt multi-center designs and RI-CLPM models to separate trait-like stability from dynamic mediation processes (93). Additionally, integrating objective SES indicators (e.g., neighborhood deprivation indices) and advanced impulsivity measures (e.g., BIS-11 cognitive subscales) would enhance validity.

## 5 Conclusion

In summary, the findings of this study reveal a significant association between childhood SES and the mental health of first-year college students, with impulsivity playing a partially conditional effect role. Furthermore, higher social support is associated with attenuated negative relationships between low childhood SES and students’ mental health, potentially through its correlation with lower impulsivity levels. Future prevention and intervention measures should emphasize the importance of controlling impulsivity and having strong social support in promoting the mental health of first-year college students.

## Data Availability

The original contributions presented in the study are included in the article. Further inquiries can be directed to the corresponding author/s.
